# Outer membrane proteins can be simply identified using secondary structure element alignment

**DOI:** 10.1186/1471-2105-12-76

**Published:** 2011-03-17

**Authors:** Ren-Xiang Yan, Zhen Chen, Ziding Zhang

**Affiliations:** 1 State Key Laboratory of Agrobiotechnology, College of Biological Sciences, China Agricultural University, Beijing 100193, PR China; 2 Bioinformatics Center, College of Biological Sciences, China Agricultural University, Beijing 100193, PR China

## Abstract

**Background:**

Outer membrane proteins (OMPs) are frequently found in the outer membranes of gram-negative bacteria, mitochondria and chloroplasts and have been found to play diverse functional roles. Computational discrimination of OMPs from globular proteins and other types of membrane proteins is helpful to accelerate new genome annotation and drug discovery.

**Results:**

Based on the observation that almost all OMPs consist of antiparallel β-strands in a barrel shape and that their secondary structure arrangements differ from those of other types of proteins, we propose a simple method called SSEA-OMP to identify OMPs using secondary structure element alignment. Through intensive benchmark experiments, the proposed SSEA-OMP method is better than some well-established OMP detection methods.

**Conclusions:**

The major advantage of SSEA-OMP is its good prediction performance considering its simplicity. The web server implements the method is freely accessible at http://protein.cau.edu.cn/SSEA-OMP/index.html.

## Background

Outer membrane proteins (OMPs), an important class of proteins, are found in gram-negative bacteria, mitochondria and chloroplasts. Computational discrimination of OMPs from globular proteins and other types of membrane proteins is helpful to accelerate new genome annotation and drug discovery. A variety of OMP identification methods have been elegantly developed [1-27] and some web servers have also been freely accessible to the research community [2,7,13,16,21,27]. However, OMPs are difficult to be discriminated from other types of proteins, and the existing methods are not entirely satisfactory, mainly because the membrane-spanning regions of OMPs are shorter and these regions usually have higher variations in properties when compared with α-helical membrane proteins [28]. Therefore, the development of new OMP identification methods with improved performance is needed. Meanwhile, it is also hoped that new OMP identification methods will be helpful to accelerate the exploration of the sequence-structure protein landscape in OMPs.

The existing OMP predictors can be categorized through different ways. According to the adopted algorithms, the predictors can be divided into simple statistical theory- and machine learning-based methods. The highlight of simple statistical methods is that the biological meanings of the established statistical models are comprehensible. Representative statistical theory-based OMP predictors include DD [15], WED [17], WED_HFS [17], BOMP [2] and TMB-Hunt [29]. The advantage of machine learning algorithms is that they can easily incorporate different information/features into an OMP discrimination system. Some state-of-the-art machine learning algorithms (*e.g.*, Neural Network (NN) and Support Vector Machines (SVM)) have been employed to construct OMP discrimination systems [5,6]. Using amino acid composition as input, Gromiha and Suwa (2006) developed a NN-based method (NN_AAC) to identify OMPs [5]. Later, they also proposed an SVM-based OMP identification method (SVM_AAC_DPC) by adding di-peptide composition information as input, which was reported to have improved performance [6]. Although machine learning algorithms can often result in excellent performance, some of them are often criticized and labeled as "black box" methods, due to a lack of biological interpretation.

According to the type of input data, the previous predictors can be roughly divided into global amino acid composition- and local sequence features-based methods. For instance, DD, WED and TMB-Hunt mainly used the global amino acid composition as input to identify OMPs. As a typical local sequence features-based method, BOMP employs C-terminal pattern recognition and a sliding window analysis of amino acid composition in alternating positions to identify OMPs. In 2005, Gromiha also proposed a local sequence features-based method, which used frequently occurring motifs to predict OMPs [11]. Moreover, the established OMP predictors can be grouped in terms of the prediction output type. Many OMP predictors such as DD, WED, WED_HFS, NN_AAC, TMB-Hunt and BOMP were limited to distinguish OMPs and non-OMPs, while a few OMP predictors (*e.g.*, PRED-TMBB [20,21], TMBETA-NET [23], TBBPred [22] and PROFtmb [30]) were also able to detect transmembrane β-strand regions in query sequences. The existing predictors could also be classified according to whether they use sequence evolutionary information or not. PRED-TMBB, DD, NN_AAC and SVM_AAC_DPC are typical methods that do not use sequence evolutionary information, while profile-based methods such as PROFtmb, HHomp are heavily relied on the use of evolutionary information. PROFtmb is a profile-based Hidden Markov Model (HMM) for the prediction of transmembrane β-strand regions and discrimination between OMPs and non-OMPs. To predict whether a query sequence is an OMP, HHomp builds a profile HMM for a query sequence and compares it with an OMP database by pairwise HMM comparison (*i.e.*, HMM-HMM matching).

Based on the observation that an OMP usually contains 8-24 antiparallel β-strands that form a barrel shape, we propose a simple method to identify OMPs using secondary structure element alignment (SSEA). Predicted secondary structure has long been known to be informative in protein structure prediction and classification. SSEA was initially proposed by Przytycka *et al. *and used as a protein taxonomy method [31]. Subsequently, SSEA has been employed as an important component for fold recognition methods such as MANIFOLD [32], GenTHREADER [33,34] and DescFold [35,36]. SSEA was also used to target new folds [37]. In our previous work, the alignment score resulting from SSEA was used as a descriptor to detect TIM-barrel proteins [38]. Additionally, Fontana and co-workers developed a web server that implements the SSEA algorithm, which is freely accessible at http://protein.cribi.unipd.it/ssea/[39]. It should also be clearly pointed out that the information of predicted secondary structure has been incorporated into some OMP predictors [7,8,19]. For instance, Liu and co-workers (2003) [8] found that the transmembrane β-strand regions in OMPs have different amino acid composition compared with the β-strands of globular proteins. Therefore, they proposed a predictor based on the composition of selected amino acids (*i.e.*, Gly, Val, Ile, Lys, Cys and Asn) in predicted β-strands to identify OMPs.

In this study, we explore a new application of SSEA by proposing an SSEA-based OMP detection method (SSEA-OMP). The proposed method was intensively tested against well-established OMP discrimination methods and found to be very competitive, suggesting that it can serve as a useful tool to assist in proteome-wide OMP annotation.

## Results and discussion

### Overall performance of SSEA-OMP

Based on the GS-dataset [15], the overall performance of SSEA-OMP was assessed by a Leave-One-Out (LOO) test. It is worth mentioning that some protein pairs in the GS-dataset may share more than 25% sequence identity. More detailed information of GS-dataset can refer to Methods section. To avoid performance overestimation caused by redundant proteins, we employed a stringent sequence-filtering method in each step of the LOO test, which can be divided into two procedures. In the first procedure, significant sequence similarity between the test protein and the library can be removed. Briefly, only the remaining sequences sharing a sequence identity (global alignment mode inferred from the algorithm of Needleman and Wunsch [40]) less than 25% and a BLAST *e*-value greater than 0.01 with the test protein were kept in the sequence library. Even with the above filtering procedure, the sequence profiles of the remaining sequences in the library, which were required by PSIPRED [41], may still contain some sequence members sharing significant homology with the test protein. Therefore, in the second filtering procedure we further filtered the remaining sequences by using the PSI-BLAST [42]*e*-value. Briefly, all the sequence profiles of the remaining sequences in the library were jumpPSI-BLASTed against the test protein. Only the remaining sequences with a PSI-BLAST *e*-value greater than 0.01 with the test protein were further kept. Based on the above two procedures, we can guarantee that the sequence similarity between the test protein and the filtered library should be very low.

**Figure 1:**
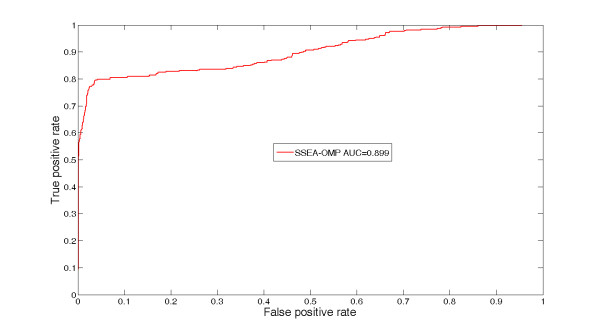
**ROC curve of SSEA-OMP assessed using the GS-dataset**. The overall performance of SSEA-OMP was assessed by the Leave-One-Out (LOO) test. In each step of the LOO test, only the remaining sequences sharing a sequence identity less than 25%, a BLAST *e*-value greater than 0.01 and a PSI-BLAST *e*-value greater than 0.01 were kept and used as the training dataset.

As shown in Table 1, SSEA-OMP resulted in an excellent performance. Four measurements, *i.e.*, Accuracy (*Ac*), Sensitivity (*Sn*), Specificity (*Sp*) and Matthew correlation coefficient (*MCC*), were jointly used to assess the performance of different methods. In general, *MCC *is a more suitable parameter than *Ac *for assessing the two-class prediction when the numbers of samples in the two classes are not equal. The value of *MCC *was therefore considered the main measurement by which to assess the performance of SSEA-OMP in this work. For instance, the proposed SSEA-OMP can distinguish between OMPs and non-OMPs with an *MCC *value of 0.772 (*Ac *= 90.9%, *Sn *= 72.9% and *Sp *= 98.1%). The overall performance of SSEA-OMP was further measured by an ROC curve. As can be seen in Figure 1, SSEA-OMP yields an AUC score (*i.e.*, the area under the ROC curve) of 0.899. The performance of SSEA-OMP at low false positive rates is also impressive. For instance, SSEA-OMP was able to correctly identify 62.9% OMPs at a less than 1% false positive rate (Figure 1). For comparison, the performance using only the first sequence-filtering procedure is also shown in Table 1. When only the first procedure was adopted, the *MCC *value of the proposed SSEA-OMP can be dramatically increased to 0.909 (*Ac *= 96.2%, *Sn *= 91.5% and *Sp *= 98.1%), implying the results are strongly affected by the sequence-filtering method.

**Table 1:** Performance of different OMP discrimination methods based on the GS-dataset.

Method	*MCC*	*Ac *(%)	*Sn *(%)	*Sp *(%)
DD^a,b^	0.541	82.4	78.8	83.3
NN_AAC^a,b^	0.716	91.0	79.3	93.8
SVM_AAC_DPC^a,b^	0.816	93.9	90.9	94.7
SSEA-OMP^c^	0.772	90.9	72.9	98.1
SSEA-OMP^d^	0.906	96.2	91.5	98.1

^a^DD, NN_AAC and SVM_AAC_DPC were developed in Suwa's group[5,6,15].

^b^The corresponding results are directly cited from [5,6,15].

^c^Based on the stringent sequence-filtering method. Briefly, only the remaining sequences sharing a sequence identity less than 25%, a BLAST e-value greater than 0.01 and a PSI-BLAST *e*-value greater than 0.01 with the test protein were kept in the sequence library.

^d^Only the first sequence-filtering procedure was employed. Briefly, only the remaining sequences sharing a sequence identity less than 25% and a BLAST *e*-value greater than 0.01 with the test protein were kept in the sequence library. It should be emphasized that the performance of SSEA-OMP based on the first sequence-filtering procedure could be overestimated. We list the SSEA-OMP performance based on the first sequence-filtering procedure to allow a generally fair comparison between SSEA-OMP and the other three methods, since the performance of the other three methods were characterized by simple sequence identity-based filtering procedure [5,6,15].

### Comparison with existing OMP discrimination methods

In this work, SSEA-OMP was intensively tested against some existing predictors. To make a fair and comprehensive comparison, we tested SSEA-OMP using two established benchmark datasets (the GS-dataset and the R-dataset [7]). Because the performance of some existing methods using these two datasets has been reported in the literature, the corresponding results were used to make a direct comparison with our SSEA-OMP.

Developed in Suwa's group, the DD, NN_ACC and SVM_ACC_DPC methods have been benchmarked in the GS-dataset, which allows us to compare SSEA-OMP with them directly (Table 1). It should be emphasized that the performance of DD, NN_ACC and SVM_ACC_DPC was evaluated in the GS-dataset without any stringent sequence-filtering procedure [5,6,15]. For instance, SVM_AAC_DPC [6] only used CD-HIT [43] to ensure that the sequence identity between any two sequences in GS-dataset could be less than 40%. As pointed out in the previous section, the performance of SSEA-OMP is strongly affected by the sequence-filtering method. Even with the same benchmarking dataset, the comparison of different methods should ideally be conducted under the same sequence-filtering method. To have a comparatively fair assessment, we benchmarked SSEA-OMP against these three methods based on the performance of SSEA-OMP when only the first sequence-filtering procedure was used. As shown in Table 1, SSEA-OMP performs better than the DD, NN_ACC and SVM_ACC_DPC methods.

**Figure 2:**
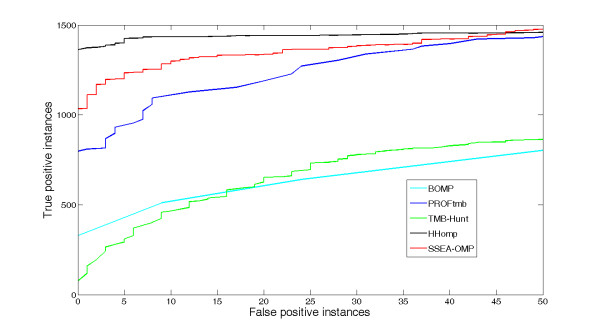
**ROC curves of different OMP discrimination methods assessed using the R-dataset**. The ROC curves of HHomp, PROFtmb, BOMP and TMB-Hunt were previously reported by Remmert *et al. *(2009) [7] and the corresponding data points were downloaded from ftp://ftp.tuebingen.mpg.de/pub/protevo/HHomp/benchmark/. To benchmark the overall performance of SSEA-OMP on the R-dataset, we also used the stringent sequence-filtering method. After the sequence filtering in each step of the benchmark experiment, we ensured that any sequence in the reference dataset should share a sequence identity less than 25%, a BLAST *e*-value greater than 0.01 and a PSI-BLAST *e*-value greater than 0.01 with the test sequence in the R-dataset.

SSEA-OMP was also benchmarked against HHomp based on the R-dataset. As reported by Remmert *et al. *(2009), HHomp used 486 OMP sequence clusters ftp://ftp.tuebingen.mpg.de/pub/protevo/HHomp/db/HHompDB_1.0.hhm, which were derived from 23 structurally solved OMPs, as the reference dataset. Furthermore, the performance of HHomp was evaluated on the R-dataset. To allow an impartial comparison between SSEA-OMP and HHomp, we compiled 486 consensus sequences from the 486 sequence clusters and the non-OMPs in the GS dataset into a library. Thus, the comparison of SSEA-OMP and HHomp was based on the reference datasets with the same OMPs. To strictly test the R-dataset, the stringent sequence-filtering method used in the LOO test of the GS-dataset was also employed. After the sequence filter in each step of the benchmark experiment, we ensured that sequences kept in the library should share a sequence identity less than 25%, a BLAST *e*-value greater than 0.01 and a PSI-BLAST *e*-value greater than 0.01 with the test sequence in the R-dataset. In the original paper of HHomp [7], the performance of BOMP http://www.bioinfo.no/tools/bomp, TMB-Hunt http://bmbpcu36.leeds.ac.uk/~andy/betaBarrel/AACompPred/aaTMB_Hunt.cgi and PROFtmb [30] based on the R-dataset was also assessed by directly submitting the sequences in the R-dataset to the corresponding OMP prediction servers, which also facilitates us to compare SSEA-OMP with these peer OMP predictors. Because the performance at low false positive rates is more important for real-world application, here we pay more attention on comparing difference methods' performance at low false positive rates. For instance, SSEA-OMP correctly recognizes 1036 OMPs before including the first false positive, whereas HHomp, BOMP, TMB-Hunt and PROFtmb can detect 1363, 329, 76 and 798 OMPs, respectively (Figure 2). At a less than 1% false positive rate (*i.e.*, 50 false positive instances) control, SSEA-OMP can correctly recognize 1476 OMPs, which is slightly better than the performance of HHomp and PROFtmb (1458 and 1429, respectively) and significantly higher than the correctly identified numbers of TMB-Hunt and BOMP (862 and 641, respectively) (Figure 2).

Based on the above benchmark experiments, SSEA-OMP has showed a fully comparable performance to some existing OMP predictors. Although many efforts were taken to make sure that the above benchmark experiments were intensive and strict, it should be pointed that we are still not able to guarantee a fully unbiased assessment. We take the performance comparison in the R-dataset as an example to discuss the potential biases. To obtain the performance of BOMP, PROFtmb and TMB-Hunt, Remmert *et al. *(2009) submitted the R-dataset directly to the corresponding web servers. On the one hand, some proteins in the R-dataset are likely to have been used to training the BOMP, TMB-hunt and PROFtmb servers. Therefore, the performance of these three methods might have been overestimated. On the other hand, the training/reference datasets in these three OMP predictors are not fully identical, although all of them are based on known OMPs with experimentally solved 3D structures. Generally, a training dataset covering a more complete sequence/structure space of known OMPs should result in a more favorable performance. Thus, the comparison bias among these three predictors has also been inevitably caused by the corresponding training datasets. With more and more OMP predictors available to the community, critical assessment of different predictors based on some standard training and test datasets is increasingly important. We hope such datasets will be available in the near future. Thus, different OMP predictors can be more reliably benchmarked. Meanwhile, initiatives in establishing some real-time comparison methods by following similar strategies in assessing different protein structure prediction methods (*e.g.*, Live-Bench [44] and EVA [45]) should also be helpful to advance the method development of OMP identification.

### Benchmark experiment on β-class globular proteins

Since all-β globular proteins and OMPs may have high similarity in secondary structure arrangements, one may argue that all-β proteins should have higher probability to be predicted as OMPs and this could be a limitation of SSEA-OMP. To investigate whether all-β proteins tends to be predicted as OMPs in our SSEA-OMP, the following benchmark experiment was carried out. We relied on the SCOP database (version 1.75) to compile a large-scale benchmarking dataset. The SCOP_1.75_40% dataset with a total of 10567 proteins was first downloaded from the ASTRAL website http://astral.berkeley.edu/, in which the sequence identity among the proteins is equal to or less than 40%. Only the sequences in the four major globular protein classes (*i.e.*, all-α, all-β, α/β and α+β proteins) were kept. For each fold in all-α, α/β and α+β proteins, only one representative sequence was further kept. Thus, we compiled a dataset called SCOP_1.75_3000, which contains 2197 all-β proteins and 803 non-all-β proteins. Using the GS-dataset as the library, the SCOP_1.75_3000 dataset was processed by SSEA-OMP. In each step of the benchmark experiment, a BLAST sequence-filtering method was applied to ensure that only the sequences sharing a BLAST *e*-value greater than 0.01 with the test protein were kept in the library. Of the 2197 β-class proteins, 63 proteins were predicted as OMPs (*Sp *= 97.1%). Concerning the prediction of 803 non-all-β proteins, 16 of them were predicted as OMPs (*Sp *= 98.0%). Therefore, SSEA-OMP does not show a tendency to generate significantly higher false positive rate in predicting all-β proteins as OMPs, implying the secondary structure topology difference between OMPs and all-β proteins (*e.g.*, the number of β-strand elements, the order of secondary structure elements and the length of each element) can be sensitively captured by SSEA.

### The web server

To aid the research community, a web server that implements the SSEA-OMP method was constructed and is freely accessible at http://protein.cau.edu.cn/SSEA-OMP/index.html. The GS-dataset is used as the library for our web server. For a query sequence, the web server returns the top hit to OMPs and the corresponding SSEA similarity and prediction scores. Moreover, the alignment between secondary structural elements of the query sequence and the top hit is provided. It was estimated that a prediction score ≥0.021 yields a false positive rate of ≤1% (*i.e.*, 99% confidence level). Currently, a four-CPU Dell Linux machine with 16 GB of main memory hosts the web server. The multi-thread technique was employed and the computational time required for processing a query sequence of 600 amino acids is usually not more than two minutes.

It should be pointed out that SSEA-OMP's performance is related to the selected library to some extent, which has been clearly reflected in the aforementioned two benchmark experiments. Similar to some other OMP predictors such as HHomp [7], one major limitation of SSEA-OMP is that it can only identify OMPs sharing similar secondary structure topology with known OMPs in the library. Experienced users may prefer to download our in-house SSEA algorithm http://protein.cau.edu.cn/pdbs/SSEA.tar.gz and use a library compiled by themselves for some proteome-wide OMP identification tasks.

### Proteome-wide OMP identification in *Escherichia coli*

To provide a practical application of SSEA-OMP, we conducted a proteome-wide OMP identification in *E. coli*. The whole proteome of *E. coli*, which contains 4126 protein sequences, was downloaded from the NCBI website ftp://ftp.ncbi.nih.gov/genomes/Bacteria/Escherichia_coli_K_12_substr__DH10B_uid58979/. All the *E. coli *protein sequences were directly submitted to the SSEA-OMP web server and 167 proteins were predicted to be OMPs with a false positive rate control of 1% (Additional file 1).

To assess the performance of SSEA-OMP, we collected known *E. coli *OMPs from public databases. In fact, there are 74 proteins annotated as OMPs in the downloaded *E. coli *proteome dataset. 94 *E. coli *proteins in the PSORTdb database [46] are characterized as OMPs through experimental study or computational prediction, and 69 *E. coli *proteins are also annotated as OMPs in the OMPdb database [47]. We extracted all OMP annotations from these three datasets, and compiled a total number of 120 proteins into a known *E. coli *OMP dataset. Of the 167 predicted OMPs, 80 proteins have been included in the known *E. coli *OMP dataset. Therefore, these 80 predicted OMPs should be regarded as true positives with high confidence. Since it is estimated that OMPs consist of 2-3% of the complete proteomes [48], the current OMP prediction apparently resulted in a certain number of false positives. When further searched the PSORTdb database, 55 hits are clearly annotated as non-OMPs in terms of subcellular localization information, suggesting that they are very likely to be false positives. The remaining 32 proteins, whose subcellular localizations are annotated as "unknown" or "this protein may have multiple localization sites" in the PSORTdb database, may be the potential OMPs that have not been previously discovered.

Considering the highly imbalanced numbers of OMPs and non-OMPs in a proteome, it is not surprising that our SSEA-OMP resulted in a certain number of false positives even at a false positive rate control of 1%. In order to reduce the false positives, we may resort to other bioinformatics tools. For example, false positive predictions could be further reduced employing a signal peptide predictor (*e.g.*, SignalP server [49]) according to the fact that most OMPs have a signal peptide [20,46]. Alternatively, we may choose the threshold value at a higher confidence level, but the identified true positives will be reduced accordingly. It should also be mentioned that 39 known *E. coli *OMPs were not successfully identified by SSEA-OMP, which may be ascribed to the fact that some of these 39 proteins share dissimilar secondary structure topology with known OMPs in the SSEA-OMP library. To maximize the performance of SSEA-OMP, a regularly-updated library which covers all sequence/structure space of known OMPs is highly desired.

## Conclusions

Taking together these findings, we have clearly demonstrated that OMPs can be simply identified using SSEA-OMP. First, the success of SSEA-OMP should be ascribed to the facts that known OMPs have similar secondary structure topologies and the overall similarity of secondary structure topology between two OMPs can be sensitively detected by SSEA. Although predicted secondary structure has been incorporated into several existing OMP predictors [7,8,19], it should be emphasized that our SSEA-OMP utilizes the information of secondary structure in a different way. Second, the high accuracy of SSEA-OMP also implies that PSIPRED has reached a reasonably high degree of accuracy in predicting the secondary structure of OMPs, even though PSIPRED was not optimized for OMPs. Concerning future development, two aspects should be taken into account. First, optimization of the SSEA scoring scheme may help to improve the current version of SSEA-OMP. Second, SSEA can be used as a key feature in the construction of a new OMP discrimination method. It is hoped that the integration of SSEA with some other well-recognized features can result in a more powerful OMP discrimination system with the assistance of statistical or machine learning methods.

## Methods

### Datasets

We relied mainly on Gromiha and Suwa's dataset [15], which we refer to as the GS-dataset, to construct SSEA-OMP. The GS-dataset consists of 377 OMPs, 674 globular proteins, and 268 inner membrane proteins (IMPs). OMP discrimination can be assigned as a binary classification problem. The 377 OMPs were considered positive instances, and 942 non-OMPs (*i.e.*, the 674 globular proteins and 268 IMPs) were considered negative instances.

To critically benchmark SSEA-OMP against some of the existing methods, the R-dataset [7], which contains 2164 OMPs from the TransportDB database [50] and 5000 non-OMPs randomly selected from the SCOP database (version 1.69) [51], was downloaded from ftp://ftp.tuebingen.mpg.de/pub/protevo/HHomp/benchmark/.

The NCBI non-redundant (NR) sequence database (November 2008 version) was downloaded from ftp://ftp.ncbi.nlm.nih.gov/blast/. The NR database was further clustered at 90% sequence identity (global alignment mode) by using CD-hit [43], and the resulting NR90 database was used to implement the PSI-BLAST search [42].

### Construction of SSEA-OMP

To develop the SSEA-OMP method, our in-house SSEA algorithm was used. Performing an SSEA for two sequences typically consists of three steps. First, the two sequences were PSI-BLASTed against the NR90 database for three iterations to generate the corresponding PSSM profiles. The *e*-value cutoff for including sequences in the PSSM profiles was set at 0.001. The obtained PSSM profiles were further employed as input to PSIPRED [41] to predict the secondary structures of the two sequences. Second, the predicted secondary structural string for each sequence was converted into secondary structure elements such that "H" represents a helix element, "E" denotes a strand element, and "C" stands for a coil element. Thus, the predicted secondary structural string was shortened and the length of each element was retained for the scoring of SSEA. For example, the secondary structure string HHHHCCCCCEEEEEHHHH would be shortened to HCEH, and the lengths of all elements (*i.e.*, 4, 5, 5 and 4) would be stored. Third, the two shortened strings were aligned using a modified dynamic programming algorithm with a scoring scheme adopted from Przytycka *et al. *(1999) [31]. The detailed alignment score between two elements with lengths *L*_*i *_and *L*_*j *_is defined as:(1)

where min(*L*_*i*_, *L*_*j*_) stands for the minimal length between *L*_*i *_and *L*_*j*_. Additionally, the gap cost in SSEA is set to 0. Since the alignment score between two elements is equal to or less than min(*L*_*i*_, *L*_*j*_), the total alignment score between two sequences is equal to or less than the minimal sequence length of these two sequences. To obtain a normalized SSEA similarity score (*SSEA_Score*), the total alignment score is further divided by the average length of these two sequences. Therefore, the *SSEA_Score *is always in the range of 0 to 1. Generally, the closer the SSEA similarity score is to 1, the more significant is the secondary structure-based similarity between two sequences. In the original SSEA algorithm [31] both helix and strand elements can be split for alignment with coil elements, and coil elements can be split into either two or three smaller coil elements. However, Fontana *et al. *(2005) [39] argued that the partition of secondary structure elements can not obtain better alignment between two proteins. Therefore, we do not split any secondary structure element in our SSEA algorithm.

With the established SSEA algorithm, SSEA-OMP can be easily implemented by selecting a suitable sequence library (*i.e.*, reference dataset). In this work, we used the GS-dataset as the sequence library. For a query sequence, SSEA-OMP calculated the pair-wise SSEA similarity scores between the query sequence and all proteins in the GS-dataset. The top hit to OMPs and the corresponding SSEA similarity score (*i.e., SSEA_Score*_*max,OMP*_) were recorded. Meanwhile, the maximal SSEA similarity score between non-OMPs in the library and the query protein (*i.e., SSEA_Score*_*max,non-OMP*_) was also calculated. For a query sequence, a prediction score (*i.e., Pred_Score*) was defined as:(2)

The query protein was predicted to be an OMP if *Pred_Score *> 0; otherwise, it was predicted to be a non-OMP.

### Performance assessment of SSEA-OMP

The performance of SSEA-OMP was assessed using the LOO test. For each step of the assessment, a protein in the GS-dataset was selected as a test protein, and the remaining proteins were regarded as a sequence library. The test protein was scanned against the sequence library using SSEA, and the prediction result was recorded. When the test was performed over all proteins in the GS-dataset, the overall performance of SSEA-OMP was evaluated with respect to four parameters: *Ac*, *Sn*, *Sp *and *MCC*. These parameters are defined below:(3)(4)(5)(6)

where *tp*, *fp*, *fn *and *tn *denote true positives, false positives, false negatives and true negatives, respectively. The performance of an OMP discrimination method can also be systematically measured by a receiver operating characteristic (ROC) curve. Using a strategy similar to the one reported by Yan *et al. *(2008) [17], we defined a threshold parameter, α, such that the test protein was predicted to be an OMP if *Pred_Score *> α. The ROC curve of SSEA-OMP plots true-positive instances as a function of false-positive instances for all possible values of α. Additionally, the ROC curve can be further quantified by the AUC score, which represents the area under the ROC curve.

## Availability and requirements

**Project Name**: SSEA-OMP

**Project home page**: http://protein.cau.edu.cn/SSEA-OMP/index.html

**Operating system**: Online service is web based; local version of the software is platform independent.

**Programming language**: Perl and Java.

**Other requirements**: None.

**License**: Free.

**Any restrictions to use by non-academics**: None.

## Authors' contributions

RXY wrote the codes, developed the web server, and drafted the manuscript. ZC participated in the method assessment. ZZ directed the research and revised the manuscript. All authors read and approved the final manuscript.

## Supplementary Material

Additional file 1 **is a text file showing the predicted 167 *E. coli *OMPs**. Click here for file
